# Role of metabolic reprogramming and lactylation in diabetic nephropathy: molecular mechanisms and therapeutic prospects – a narrative review

**DOI:** 10.3389/fendo.2026.1770531

**Published:** 2026-02-13

**Authors:** Zongtao Li, Yashi Wang, Die Fang, Wenfeng Ye, Xueqin Zhang, Zhiqiang Chen

**Affiliations:** Hebei University of Chinese Medicine, Shijiazhuang, China

**Keywords:** diabetic nephropathy, epigenetic regulation, glycolysis, lactate accumulation, lactylation, metabolic reprogramming, mitochondrial dysfunction

## Abstract

Diabetic nephropathy (DN) remains challenging to halt completely despite standard therapies. Beyond being a glycolytic byproduct, lactate can act as a signaling metabolite and a substrate for lysine lactylation, linking metabolic reprogramming to chromatin regulation. To summarize the lactate–lactylation axis in DN and its translational implications, we conducted a narrative review by searching PubMed, Web of Science, and Scopus for English-language studies up to October 2025 using terms related to DN, metabolic reprogramming, glycolysis, lactate, and lactylation. Evidence was prioritized from DN/kidney studies, with selected mechanistic reports from related contexts included when relevant. The results show that hyperglycemia, hypoxia, and inflammation promote a shift toward glycolysis, leading to lactate accumulation, mitochondrial dysfunction, and inflammatory signaling in DN. Lactate-associated histone and non-histone lactylation has been implicated in cell-type–dependent transcriptional programs across tubular epithelial cells, podocytes, endothelial cells, and immune cells, potentially amplifying inflammation and fibrosis. Candidate “writers/erasers” and emerging non-canonical lactyltransferase activities suggest lactylation may represent a tunable epigenetic node. Thus, the lactate–lactylation axis provides a promising but evolving framework for DN pathogenesis and therapy. Future work should prioritize DN-focused validation, including studies using human DN samples where feasible, standardized detection, and stage- and cell-specific interventions to minimize systemic metabolic disruption.

## 1 Introduction

Diabetic nephropathy (DN) is one of the most common and destructive microvascular complications of diabetes and the leading cause of end-stage renal disease (ESRD) worldwide (1, 2). With the increasing incidence of type 2 diabetes worldwide, the prevalence of DN is also rising. Although improvements in blood glucose management and the inhibition of the renin-angiotensin-aldosterone system (RAAS) have to some extent alleviated the condition, a considerable proportion of patients still experience continuous deterioration of renal function (3). A major challenge is that current therapies reduce risk but rarely eliminate residual progression, particularly once chronic inflammation and fibrotic remodeling become established across multiple renal cell types. This residual risk suggests that DN progression is driven by intertwined metabolic, inflammatory, and fibrotic programs rather than a single pathway.

Current research indicates that the development of DN is not caused by a single factor, but is the result of the combined action of multiple mechanisms such as metabolic disorders, inflammatory responses, and fibrotic remodeling. Among them, metabolic reprogramming has become one of the notable features of disease progression. Renal cells gradually shift from a mitochondria-dependent oxidative phosphorylation (OXPHOS) mode to enhanced glycolysis. This metabolic alteration leads to the continuous accumulation of lactate, redox imbalance, mitochondrial dysfunction, and the activation of multiple inflammatory signaling pathways (4, 5). Clinical studies have also found that the urine lactate level of DN patients is significantly elevated (6), suggesting that lactate may be an important molecular link between metabolic disorders and kidney damage.

For a long time, lactate has been largely regarded as merely a terminal metabolite of glycolysis metabolism. However, in 2019, Zhang et al. discovered that lactate could serve as a substrate for a new post-translational modification-lysine lactylation (Kla) (7). This discovery directly demonstrated for the first time that metabolic intermediates can regulate gene transcription, thereby establishing a mechanistic link between metabolism and epigenetic modifications (8). Compared with epigenetic marks widely discussed in DN (e.g., acetylation and methylation), lactylation is uniquely positioned to sense glycolytic flux because it is directly coupled to lactate availability. Since then, lactate has been re-recognized as a signaling molecule that can influence cell fate and disease progression through lactylation-dependent transcriptional and functional remodeling.

**Figure 1:**
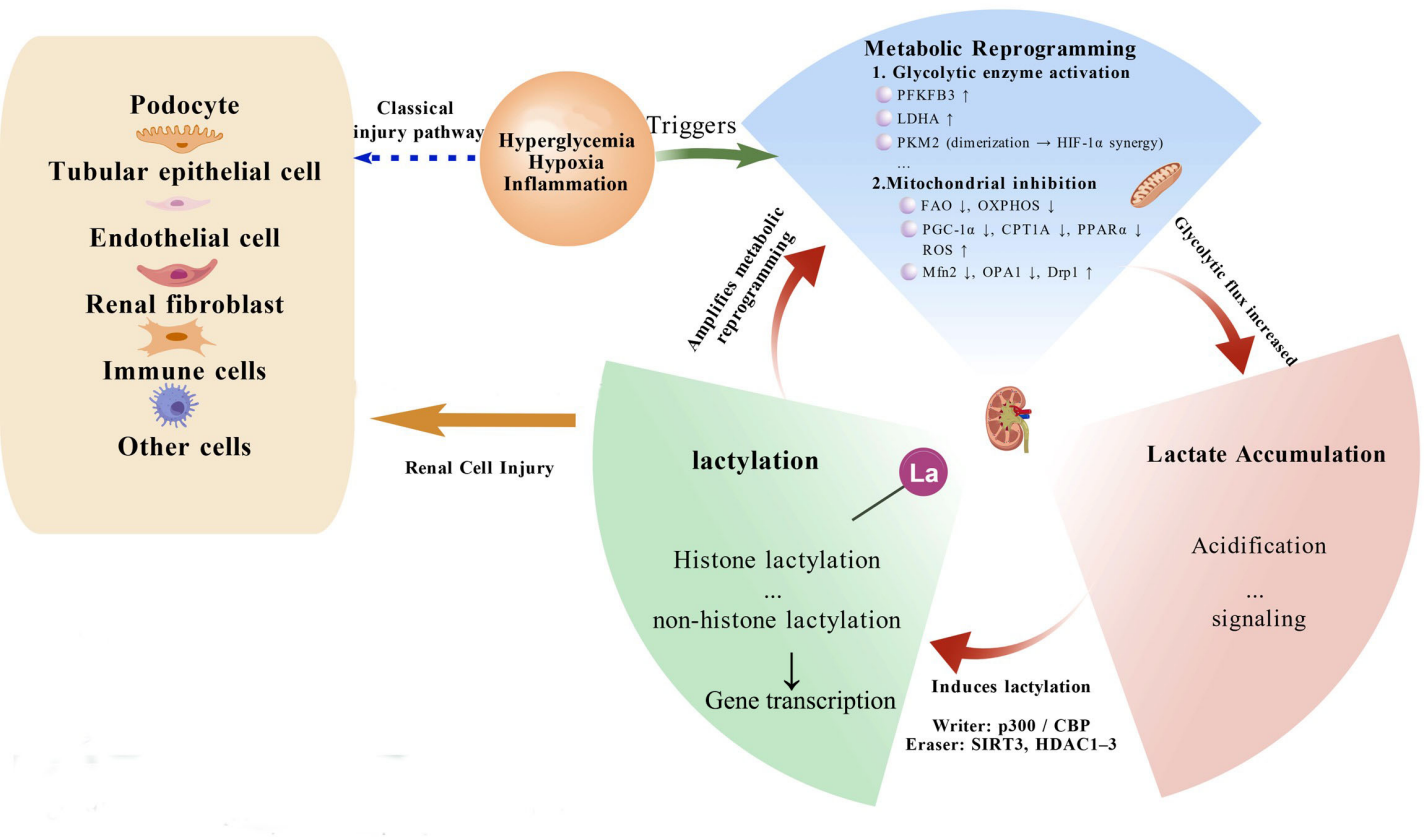
The metabolic–epigenetic feedback loop in DN. Chronic hyperglycemia, hypoxia, and inflammation collectively induce glycolytic reprogramming while suppressing mitochondrial OXPHOS, leading to excessive lactate accumulation. Lactate serves as both a signaling molecule and a substrate for lysine lactylation, which further enhances glycolytic enzyme expression and inhibits mitochondrial metabolism, forming a self-reinforcing metabolic–epigenetic loop that promotes renal inflammation, fibrosis, and cellular injury. *Abbreviations:* OXPHOS, oxidative phosphorylation; FAO, fatty acid oxidation; LDHA, lactate dehydrogenase A; HIF-1α, hypoxia-inducible factor 1α.

Based on the existing evidence, the progression of DN may involve a self-amplifying metabolic–epigenetic feedback loop, namely “metabolic reprogramming–lactate accumulation–aberrant lactylation–metabolic dysregulation and renal injury” (**Figure 1**). During this process, enhanced glycolysis promotes lactate accumulation, while lactate-induced protein lactylation further stimulates glycolytic activity and inhibits OXPHOS, thereby providing a conceptual bridge from metabolic reprogramming to chromatin/transcriptional remodeling in DN. This cycle between epigenetic modifications and metabolic disorders reveals a new mechanism framework for the pathological development of DN. Unlike previous reviews that mainly focused on metabolic reprogramming or epigenetic dysregulation, our article integrates these two aspects and highlights the lactate-lactylation feedback loop as a mechanistic bridge between metabolism and chromatin dynamics. This new perspective provides important insights for understanding the complex pathological process of DN and developing early diagnosis and metabolization-based treatment strategies. In the following sections, we outline metabolic reprogramming in DN and its connection to lactate accumulation, then discuss lactylation mechanisms and cell type–specific roles, and finally address therapeutic implications together with key limitations and future directions.

## 2 Methodology of this narrative review

We conducted a narrative review of the English-language literature by searching PubMed, Web of Science, and Scopus for articles published up to October 2025. The search terms included “diabetic nephropathy” or “diabetic kidney disease”, “lactylation” or “lysine lactylation”, “metabolic reprogramming”, “glycolysis” or “Warburg effect”, “lactate”, and “histone modification” (and related terms). We prioritized original experimental studies (*in vitro* and *in vivo*) and human/clinical studies when available, with a primary focus on DN and kidney-related evidence. Given that lactylation is an emerging epigenetic mechanism and direct DN/kidney evidence remains limited in some aspects, we also included selected mechanistic studies from related disease or tissue contexts when they provided insights that are biologically relevant to renal metabolic–epigenetic regulation. Relevant review articles were used to contextualize background and summarize the state of the field, whereas mechanistic statements and pathway inferences were preferentially supported by original experimental or clinical studies whenever available.

## 3 Metabolic reprogramming: the initiating driver of the vicious cycle

### 3.1 Altered glucose metabolism in DN: inducers and metabolic shift

Metabolic reprogramming is generally considered to be the result of renal cells in DN being exposed to multiple pathological environments over a long period of time. Continuous metabolic load, hypoxic stimulation and inflammatory response jointly disrupt the energy balance of cells, promoting the shift of energy metabolism from OXPHOS to glycolysis. This transformation marks the critical point of metabolic reprogramming and the initial stage of the vicious cycle of disease self-exacerbation.

Under physiological conditions, renal cells display substantial metabolic heterogeneity. Proximal tubular epithelial cells rely predominantly on mitochondrial fatty acid OXPHOS to sustain their high energy demand, whereas glomerular cells such as podocytes possess a comparatively greater glycolytic capacity that supports cytoskeletal remodeling and rapid, spatially restricted ATP supply. Although glycolysis generates less ATP per molecule of substrate, it produces ATP at a much faster rate than OXPHOS, enabling cells to rapidly buffer acute stress or fluctuations in local energy demand (9, 10). In the diabetic milieu, persistent hyperglycemia, oxidative stress, and mitochondrial dysfunction disrupt these cell-specific metabolic programs. Concurrent activation of multiple glucose-responsive pathways—including protein kinase C (PKC), the AGE–RAGE axis, and the hexosamine biosynthesis pathway—exacerbates oxidative injury and impairs mitochondrial oxidative metabolism, while further promoting glycolytic flux (11, 12). As a result, renal cells progressively shift toward a glycolysis-dominant metabolic state with increased lactate production, representing a pivotal early event in the initiation of metabolic reprogramming in DN.

In DN, the oxygen tension in both the renal cortex and renal medulla decreases significantly (13, 14). Hypoxia can stabilize the presence of hypoxia-inducible factor 1α (HIF-1α) by inhibiting the activity of prolyl hydroxylase (PHD), thereby upregulating the expression of multiple key glycolytic genes, such as glucose transporter 1 (GLUT1), lactate dehydrogenase A (LDHA), and pyruvate kinase M2 (PKM2) (15–17). This sequence of shifts significantly alters the direction of carbon flow, driving glucose metabolism toward glycolysis and promoting lactate accumulation. Kapitsinou et al. demonstrated that endothelial cell-specific PHD deletion can lead to the sustained stabilization of HIF-1α, enhanced glycolysis and lactate accumulation, thereby promoting the occurrence of renal interstitial fibrosis (18). This “hypoxia-induced glycolytic – lactate” pathway plays a core role in kidney injury. Moreover, as interstitial fibrosis progresses, excessive extracellular matrix (ECM) deposition will further impede oxygen diffusion, forming a positive feedback loop. This mechanism intensifies metabolic dysregulation and maintains a chronic hypoxic state by mutually reinforcing glycolytic activation and oxidative metabolism inhibition.

Subsequent studies have found that inflammatory signals can further amplify this pathological process. Under a high-glucose environment, immune cells are activated and produce inflammatory mediators (such as TNF-α and IL-1β), which can promote the expression of glycolysis-related genes by activating the NF-κB and Akt/mTOR signaling pathways, thereby maintaining a persistent inflammatory response. Thus, inflammation and metabolism can also form a mutually reinforcing relationship. Studies have shown that high glucose and inflammation can induce metabolic dysregulation in renal tubules and cause cell injury, while inflammatory signaling (NF-κB, Akt/mTOR) can further disturb tubular metabolic homeostasis. Modulating these pathways has been reported to affect glycolysis-related gene expression (19–21). These results suggest that inflammation is not only a result of metabolic disorders but also a positive feedback mechanism for them.

**Figure 2:**
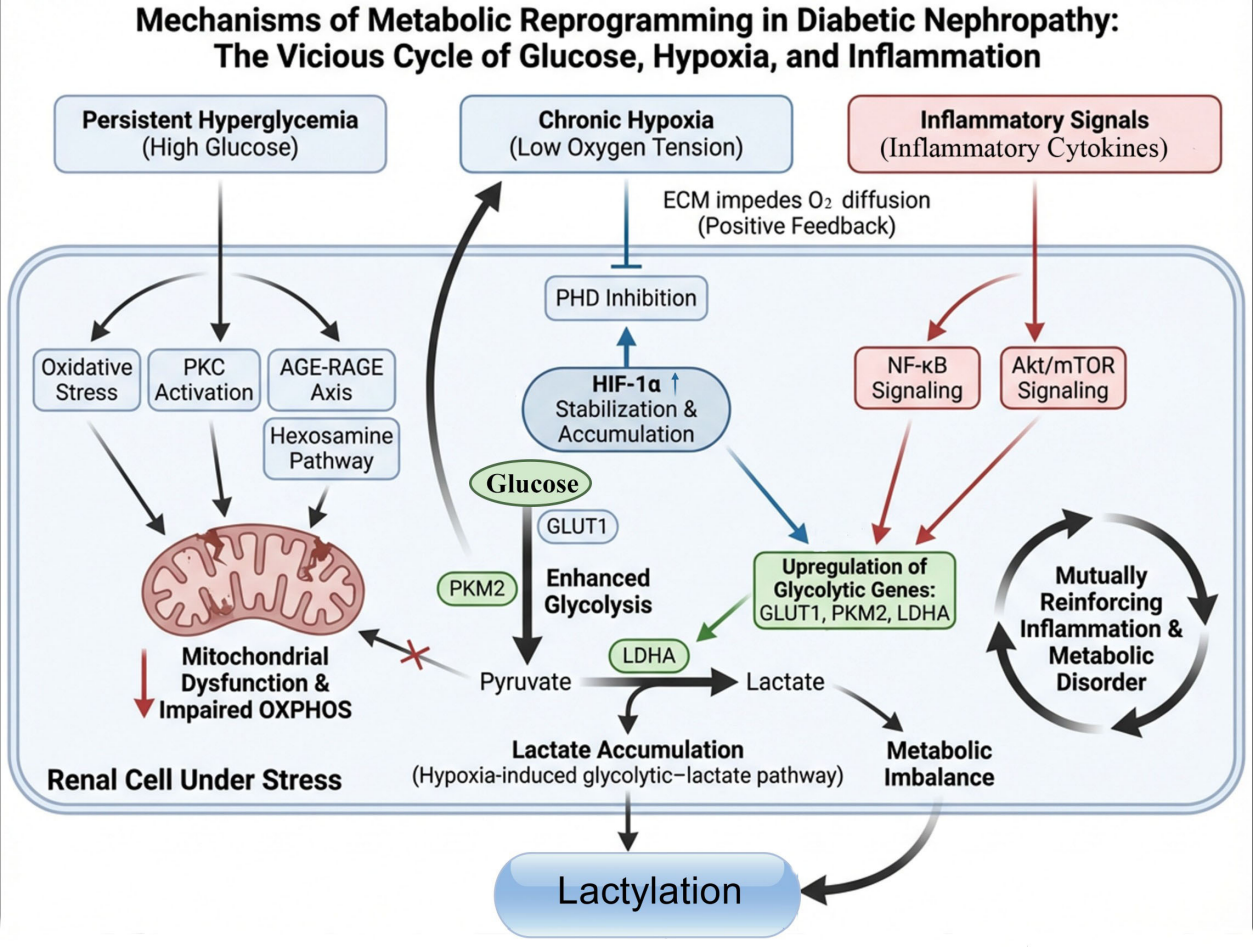
Schematic illustration of the mechanism of metabolic reprogramming in DN. Persistent hyperglycemia, chronic hypoxia, and inflammatory cytokines synergistically impose metabolic stress on renal cells, leading to mitochondrial dysfunction and impaired OXPHOS. Hypoxia-mediated stabilization of HIF-1α promotes the upregulation of key metabolic genes—including GLUT1, PKM2, and LDHA—thereby enhancing glycolysis. Meanwhile, glucose-responsive pathways (such as PKC, AGE–RAGE, and the hexosamine biosynthetic pathway) further exacerbate oxidative injury and suppress mitochondrial function. Increased glycolytic flux accelerates the conversion of pyruvate to lactate, resulting in lactate accumulation and metabolic dysregulation. Inflammatory signaling pathways additionally upregulate glycolysis-related genes. Together, these interwoven mechanisms intensify glycolytic reprogramming, generate excessive lactate, and drive epigenetic lactylation.

In summary, persistent hyperglycemia, long-term hypoxia and inflammatory signal stimulation jointly construct a self-sustaining pathological network, keeping renal cells in a metabolic state dominated by glycolysis. Conceptually, this abnormally dysregulated metabolic phenotype is considered a potential early stage of a “metabolism–epigenetic” self-reinforcing cycle driven by lactate excess and its downstream epigenetic effects (**Figure 2**), although this continuum has not yet been fully delineated in DN.

### 3.2 Shift toward aerobic glycolysis in DN

After metabolic reprogramming is initiated, due to the upregulation and enhanced activity of multiple rate-limiting enzymes and regulatory factors, the glycolytic flux of renal cells remains chronically elevated. As a result, a large amount of carbon flow from glucose is allocated to the lactate production pathway, a phenomenon that reflects the transformation of cellular energy metabolism from oxidative to glycolytic metabolism.

Among the various regulatory factors of glycolysis, 6-phosphofructo-2-kinase/fructose-2,6-bisphosphatase 3 (PFKFB3) plays a key role. PFKFB3 catalyzes the formation of fructose-2, 6-diphosphate, which is the strongest allosteric activator of phosphofructokinase-1 (PFK-1) and can significantly increase the rate of glycolytic flux. The expression of PFKFB3 in DN renal tissues was significantly upregulated, and it was jointly regulated by HIF-1α and inflammatory signaling pathways. There are also studies reporting that the expression level of PFKFB3 is positively correlated with the degree of renal interstitial fibrosis, and inhibiting PFKFB3 can alleviate inflammation and fibrotic remodeling, suggesting that PFKFB3 has a pathogenic effect in promoting the enhancement of glycolytic flux (22, 23).

Meanwhile, the expression and activity of LDHA also significantly increased in the renal tissues of DN, leading to a large accumulation of lactate within the renal tissues. This metabolic conversion reflects the continuous activation of glycolysis, and LDHA, as a key metabolic hub, links metabolic disorders with tissue damage. LDHA expression is markedly increased in DN kidneys and is associated with tissue acidosis, mitochondrial injury and accelerated fibrotic progression (24).

PKM2 is not only a metabolic enzyme but also can serve as a signal regulatory factor. Under high-glucose conditions, PKM2 dissociates from its active tetramer into a less active but transcriptionally competent dimeric form. Although the catalytic efficiency of a single dimer decreases, the dimer PKM2 can be transformed into an intracellular regulatory type and translocated to the nucleus, interacting with HIF-1α, thereby enhancing the transcription of glycolytic target genes (including GLUT1 and LDHA). Therefore, the PKM2–HIF-1α feed-forward loop further promotes glycolysis at both the metabolic and transcriptional levels (25, 26).

Together, dysregulation of key glycolytic enzymes and transcriptional regulators drives a persistent shift toward high glycolytic flux and reduced mitochondrial oxidation, functionally biasing renal parenchymal cells toward a glycolysis-dominant metabolic state.

### 3.3 Mitochondrial dysfunction

The inhibition of mitochondrial metabolism becomes a key turning point for the further amplification of the vicious cycle of metabolic disorders, in which the metabolic reprogramming of DN progresses from the stage of “enhanced glycolysis” to the stage of “systemic energy collapse”. At this point, glycolysis is highly activated, whereas the efficiency of mitochondrial OXPHOS is markedly diminished, creating a profound imbalance in cellular energy production.

At the mitochondrial level, its functional inhibition is mainly manifested as impaired fatty acid oxidation (FAO) and weakened levels of OXPHOS. FAO is the main source for maintaining energy homeostasis in proximal convoluted tubule cells, and its dysfunction is one of the early indicators of mitochondrial decline in DN, characterized by downregulation of key FAO enzymes, restricted β-oxidation, and intracellular lipid droplet accumulation. These metabolic abnormalities trigger lipotoxicity, oxidative stress, and inflammatory responses (27, 28). Studies have confirmed that activating peroxisome proliferator-activated receptor α (PPARα) can restore FAO, alleviate renal tubular injury and improve metabolic balance (29). In addition, FAO impairment can promote ubiquitination and proteasomal degradation of PPARα, exacerbating podocyte injury and proteinuria (30). These studies provide strong evidence that lipid metabolic dysfunction contributes to metabolic dysregulation across multiple renal cell types.

Beyond FAO impairment, inhibition of OXPHOS is also one of the main causes of lactate accumulation. Long-term hyperglycemia can damage mitochondrial complex I, weaken the oxidation capacity of NADH, reduce the efficiency of electron transfer and ATP synthesis, increase electron leakage and cause redox imbalance (31). To regenerate NAD^+^ and maintain glycolytic flux under conditions of impaired mitochondrial oxidation, pyruvate is converted to lactate by LDHA — a canonical biochemical step of glycolysis (10). Although this process can temporarily maintain ATP production, it simultaneously accelerates lactate accumulation and metabolic dysregulation. Meanwhile, the excessive reactive oxygen species (ROS) produced by impaired electron transport aggravates renal tubular damage by stabilizing HIF-1α and up-regulating glycolytic genes such as GLUT1, PFKFB3 and LDHA, thereby forming a positive feedback loop of “mitochondrial inhibition - HIF-1α activation - enhanced glycolytic - lactate accumulation,” thereby maintaining the continuous activation of metabolic reprogramming.

At the same time, the decline in mitochondrial function is often accompanied by a dual reduction in both quantity and quality. In DN, the main regulator of mitochondrial biogenesis PGC-1α is significantly downregulated. Part of the reason may be related to the decreased AMPK activity, resulting in limited mitochondrial renewal and impaired respiratory function. Studies have shown that overexpression of PGC-1α can restore mitochondrial abundance and function, reduce proteinuria and improve renal fibrosis (32). Additionally, mitochondrial damage is often accompanied by a severe imbalance in mitochondrial dynamics, such as overexpression of fissile protein Drp1 and down-regulation of fusion proteins Mfn2 and OPA1, which lead to excessive mitochondrial fragmentation and the generation of a large number of functionally defective organelles. These changes further reduce the oxidative capacity of mitochondria, enhance the sensitivity to apoptosis and aggravate renal tubular injury (33–35). Notably, selective inhibition of the Drp1–Fis1 interaction decreases mitochondrial fragmentation and improves renal function (36).

In summary, inhibition of mitochondrial FAO and OXPHOS not only represents a downstream result of metabolic reprogramming but also acts as an upstream amplifier that locks renal cells into glycolytic dependency. This reciprocal suppression between mitochondrial metabolism and glycolysis establishes a self-perpetuating loop that drives progressive energy collapse in DN.

### 3.4 Crosstalk between hypoxia, HIF-1α, and glycolytic enzymes

As described above, hypoxia-driven HIF-1α stabilization promotes the expression of key glycolytic genes (e.g., GLUT1, LDHA, and PKM2), thereby reinforcing glycolytic flux and predisposing to sustained lactate generation in DN. Eventually, sustained enhancement of glycolysis results in continuous lactate accumulation. Lactate is an upstream metabolomic result of DN that is consistently upregulated in animal models and human samples. Multiple studies consistently report a positive association between lactate levels and proteinuria, glomerulosclerosis, and renal function decline (37, 38). Therefore, lactate may serve not only as a metabolic byproduct but also act as an important signaling molecule in the pathogenesis of DN.

**Figure 3:**
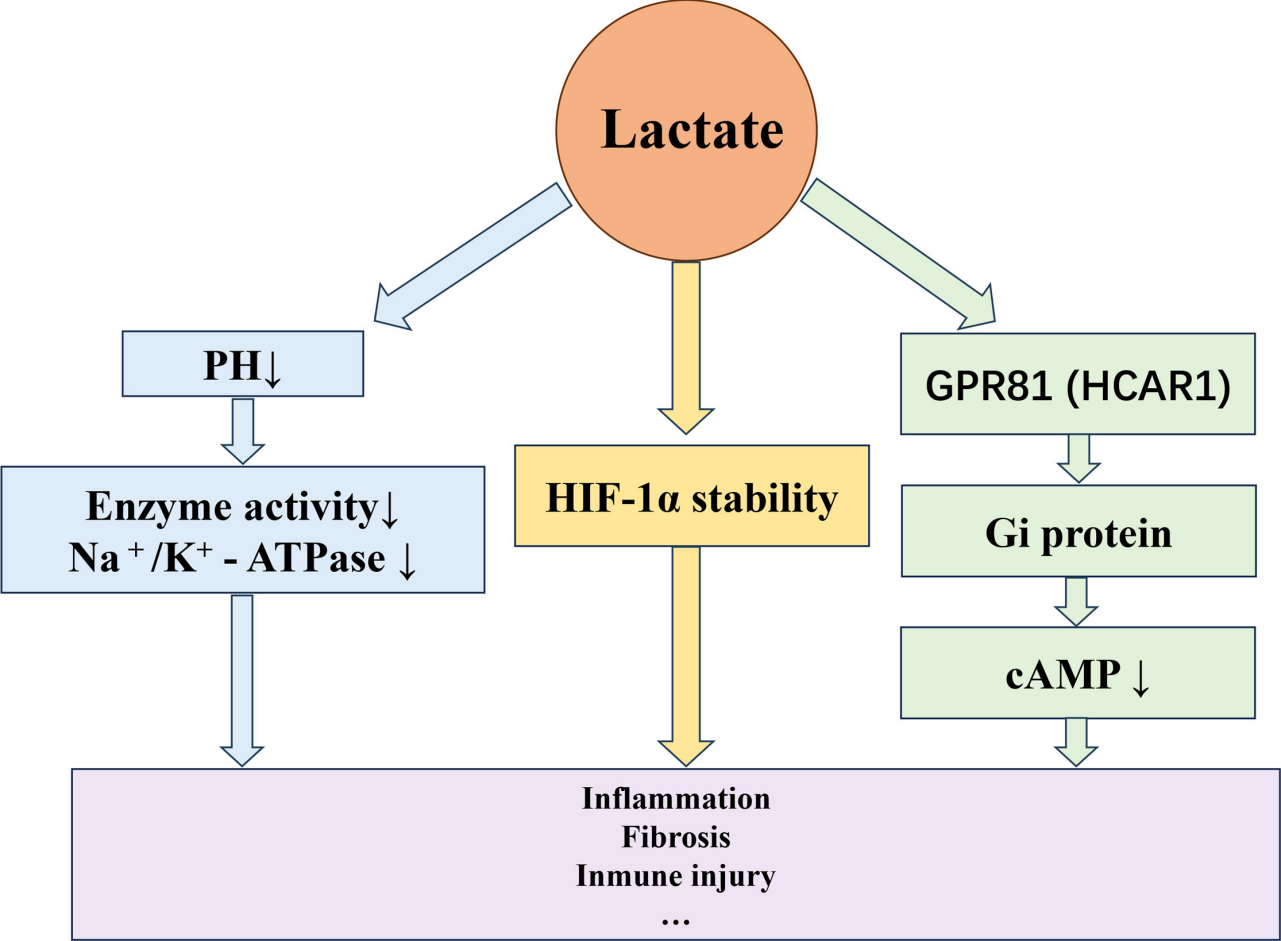
Lactate-mediated signaling and microenvironmental remodeling in DN. Accumulated lactate activates several downstream axes, including GPR81–cAMP–NF-κB–inflammatory and ROS–HIF-1α–TGF-β1/CTGF–fibrotic pathways, leading to acidification, oxidative stress, and extracellular matrix (ECM) deposition. These events disrupt renal microenvironmental homeostasis and accelerate tubular injury, vascular activation, and interstitial fibrosis. GPR81, G-protein-coupled receptor 81; ROS, reactive oxygen species; TGF-β1, transforming growth factor-β1; CTGF, connective tissue growth factor; ECM, extracellular matrix.

Lactate accumulation markedly modulates the renal microenvironment. On one hand, lactate induces tissue acidification and disturbs intracellular pH homeostasis, thereby inhibiting multiple metabolic enzymes and impairing the functions of ion channels and transporters. Lactate also acts as an endogenous ligand of GPR81 (HCAR1), activating the Gi protein–coupled signaling cascade that suppresses adenylate cyclase activity and reduces intracellular cAMP, consequently reshaping the inflammatory and metabolic status of macrophages (39, 40). In parallel, lactate has been shown to stabilize HIF-1α and drive HIF-1α–dependent macrophage polarization through a GPR81-independent mechanism (41), thereby forming a reinforcing link between lactate accumulation and HIF-1α–associated inflammatory programs. Persistent lactate-driven HIF-1α activation can subsequently amplify profibrotic pathways, including the upregulation of TGF-β1 and downstream CTGF signaling, ultimately contributing to extracellular matrix deposition and interstitial fibrosis (42–44) (**Figure 3**).

Therefore, excessive lactate accumulation serves as a critical metabolic mediator linking glycolytic reprogramming with inflammation and fibrotic signaling in DN, thereby promoting renal injury progression. Importantly, this glycolysis-biased metabolic milieu is permissive for lactylation: sustained lactate availability and proposed lactate-derived acyl donors (e.g., lactyl-CoA) may facilitate histone and non-histone lysine lactylation, providing a mechanistic basis for translating metabolic stress into chromatin-associated transcriptional programs in DN (7).

## 4 Pathological roles and mechanisms of lactylation in different renal cells

Lactate–induced lactylation may act as a bridge between metabolic regulation and epigenetic control. It can modulate chromatin activity and alter transcriptional states, thereby translating metabolic changes into gene-expression programs and linking metabolic reprogramming to pathological progression.

Intracellular lactate can be converted into lactyl-CoA, which serves as a high-energy donor for covalent modification of lysine residues under the catalysis of histone acetyltransferases (HATs). The canonical enzymes p300/CBP utilize lactyl-CoA as a substrate to catalyze histone lysine lactylation, while HDAC1–3 have been reported to exhibit histone delactylase activity in foundational studies; however, direct validation in kidney-specific cell types is still limited. In addition, SIRT3 selectively removes lactylation marks from specific histone sites such as H4K16la, indicating that the removal of lactylation is both site- and enzyme-dependent (45, 46). Together, these enzymes form a dynamic “writer–eraser” system that maintains the balance of protein lactylation.

Recent studies have found that the “writers” of lactylation are not limited to HATs. Certain aminoacyl-tRNA synthetases (aaRSs) also exhibit lactyltransferase activity. For example, alanine-tRNA synthetase (AARS1) has been identified as a novel lactyltransferase that mediates the lactylation of histone H3K18 and transcription factor STAT1 in DN models, thereby upregulating lipid metabolism–related genes and inducing lipid peroxidation and ferroptosis (47). Therefore, in addition to histones, transcription factors, metabolic enzymes, and signaling proteins are also potential targets of lactylation that can regulate their stability and activity to fine-tune cellular metabolism and signaling. Similarly, in renal carcinoma models, lactylation has been shown to stabilize the non-histone signaling protein PDGFRβ, highlighting its broader signaling regulatory potential (48).

With lactate accumulation established as a metabolic hallmark, we next focus on how lactylation may translate this metabolic signal into transcriptional and functional remodeling across renal cell types. As a novel form of metabolic signaling, lactylation directly couples the cellular energy state with transcriptional regulation. Across multiple renal cell types, high-glucose–induced metabolic reprogramming commonly leads to intracellular lactate accumulation and enhanced histone or non-histone lactylation. This lactate–lactylation axis exerts cell-specific pathological effects depending on the metabolic demands and epigenetic landscape of each cell type. The following sections outline these mechanisms in major renal cell populations. It is important to note that the strength of evidence varies between cell types, with some lactylation events directly demonstrated in DN models and others inferred from non-diabetic kidney injury or extra-renal disease models and therefore remaining hypothetical in the context of DN. A simplified working model is that high glucose promotes glycolytic flux and lactate accumulation, which can increase histone lactylation and reshape transcriptional programs, ultimately contributing to renal inflammation and fibrosis in DN. Accordingly, we interpret such findings conservatively and avoid overextending conclusions when mechanisms are mainly inferred from non-DN contexts.

### 4.1 Podocytes

Podocytes are critical structural components of the glomerular filtration barrier. Their injury is a major event leading to proteinuria and glomerulosclerosis during DN progression. Recently, it has been reported that lactate accumulation and lactate-induced lactylation may contribute to podocyte injury (38).

High-glucose conditions induce pronounced metabolic reprogramming in podocytes, including increased glycolysis, lactate accumulation, and enhanced histone lactylation. Zheng et al. found that in streptozotocin (STZ) -induced diabetic mice and the MPC5 podocyte cell line, high-glucose or exogenous lactate treatment markedly increased intracellular lactate and histone lysine lactylation (HKla) levels, while nephrin and podocin were downregulated. Moreover, α-SMA and collagen IV were upregulated, indicating that the lactate–lactylation axis could induce epithelial–mesenchymal transition (EMT) in podocytes and further impair the glomerular filtration barrier. In contrast, oxamate (a lactate production inhibitor) and dichloroacetate (DCA) (an enhancer of pyruvate oxidation) were both able to reduce lactate and HKla levels, partially restore nephrin expression, and alleviate structural injury. Consistent with these findings, DN mice treated with either agent exhibited reduced renal lactylation and improved kidney function (49).

Meanwhile, recent mechanistic studies have demonstrated that lactylation may also contribute to podocyte injury by regulating autophagy and mitochondrial stability. Fan et al. reported that LARS1 is lactylated at K970 under high-glucose conditions, which prevents its ubiquitination and proteasomal degradation (50). This lactylation modification causes LARS1 to accumulate and activates mTORC1. The continuous activation of mTORC1 inhibits autophagy function, leading to the inability to clear the damaged mitochondria within the cells. With the impairment of mitochondrial function, the intracellular ROS level increases, eventually inducing podocytes to undergo apoptosis and destroys the renal tubular filtration barrier.

### 4.2 Tubular epithelial cells

As tubular epithelial cells have the highest energy demands among all renal cell types and are highly susceptible to metabolic disorders, metabolic stress induced by lactate persists even after pathological stimuli subside (23, 51).

In proximal tubular cells, PFKFB3-driven glycolytic activation represents one of the clearest links between metabolic reprogramming and epigenetic dysfunction in DN. PFKFB3 upregulation markedly elevates glycolytic flux and lactate production, which in turn enhances histone H4K12 lactylation. This modification promotes NF-κB–dependent inflammatory transcription and exacerbates tubular injury and fibrosis. Thus, the PFKFB3–lactate–H4K12la axis serves as a representative model of how metabolic stress is translated into epigenetic dysregulation in tubular cells. Similarly, in models of acute kidney injury (AKI), lactylation marks such as H3K18la were upregulated at tubular injury sites and enriched at promoters of inflammatory genes (52), suggesting that lactylation may function as an inflammatory amplifier in renal tubular injury models.

At the metabolic signaling level, lactylation of key regulatory proteins affects energy metabolism and cell fate in tubular epithelial cells. For example, in diabetic models, lysine lactylation of ACSF2 at K182 impairs its ability to facilitate the activation of fatty acids for mitochondrial β-oxidation, leading to energy deficit, excessive ROS accumulation, and aggravated tubular injury (51). In addition, in AKI-to-chronic kidney disease (CKD) transition models, lactylation of citrate synthase (CS) facilitates the assembly of the NLRP3 inflammasome and activation of caspase-1, thereby intensifying chronic inflammation and accelerating fibrotic progression (53). More importantly, in lactate-driven tubular injury and DN models, p300-dependent lactylation of the E3 ubiquitin ligase TRIM65 at lysine 206 (K206) impairs the ubiquitin-mediated degradation of IREB2 and PDK4, leading to heightened ferroptotic sensitivity and persistent glycolytic activation. In contrast, mice expressing the non-lactylatable TRIM65-K206R mutant showed enhanced antioxidant capacity and significant renal protection (54).

In summary, lactylation acts as a key regulator that affects glycolysis, lipid oxidation, and inflammatory signaling, thereby promoting metabolic dysregulation and chronic injury in tubular epithelial cells.

### 4.3 Endothelial cells

Endothelial cells, the major constituents of the renal microvasculature, maintain microcirculatory and oxygen-metabolic homeostasis by controlling vascular permeability and local blood flow. In DN, metabolically reprogrammed endothelial cells exhibit enhanced glycolysis and lactate accumulation, which contribute to aberrant angiogenesis, endothelial-to-mesenchymal transition (EndoMT), and microvascular remodeling through lactylation-dependent mechanisms.

Lactate–mediated lactylation is critically involved in regulating endothelial angiogenesis. In an *in vitro* endothelial angiogenesis model, Fan et al. (2024) found that lactate enhances histone H3K9 lactylation, which in turn sensitizes endothelial cells to VEGF signaling and potentiates angiogenic responses (55). Under lactate exposure, endothelial cells show prominent enrichment of H3K9la at angiogenesis-related loci, indicating that lactylation acts as an epigenetic amplifier of VEGF-driven angiogenesis. Although the involvement of lactate-induced lactylation in DN-associated microvascular injury has not been directly confirmed, these findings suggest a plausible mechanism by which metabolic stress may dysregulate renal angiogenesis. Lactylation also participates in endothelial phenotypic transition. In myocardial infarction models, lactate-induced lactylation of the transcription factor Snail1 promotes EndoMT, endothelial dysfunction, and microvascular rarefaction (56). Although direct evidence in DN is currently lacking, lactylation-dependent modulation of endothelial phenotype may plausibly participate in microvascular injury, EndoMT, and the progression of interstitial fibrosis in diabetic kidneys. Although direct evidence in DN is lacking, a similar mechanism may contribute to diabetic renal microvascular injury, given the shared features of hypoxia, oxidative stress, and lactate overload across these pathological contexts.

### 4.4 Immune cells

Lactylation is an important epigenetic regulation method that changes the phenotype and function of immune cells. In the lactate-rich environment of DN, this modification serves as a metabolic–epigenetic switch that determines the direction and persistence of immune activation.

The immunoregulatory role of lactylation was first discovered in macrophages. During the resolution phase of inflammation, lactate accumulates in M1 macrophages and induces enriched histone H3K18 lactylation at promoters of M2-related genes such as ARG1, thereby promoting a shift toward an anti-inflammatory, tissue-repair phenotype (7). A similar mechanism may operate in DN, where high lactate levels may drive histone lactylation and bias macrophages toward an M2-like phenotype. These lactylated macrophages secrete profibrotic mediators including TGF-β, contributing to interstitial fibrosis progression. Inhibiting lactate production or lactylation reduces M2 accumulation and mitigates fibrotic remodeling.

In addition to polarization, lactylation also modulates immune-inflammatory signaling cascades. In a sepsis-induced AKI model, high concentrations of lactate enter macrophages through monocarboxylate transporters and induce p300/CBP-mediated lactylation of high-mobility group box 1 (HMGB1) (57). This modification enhances HMGB1 exosome secretion. Simultaneously, lactate suppresses SIRT1 activity, leading to both acetylation and lactylation of HMGB1, thereby promoting its nuclear export and amplifying systemic inflammation. Pharmacological inhibition of lactate production or GPR81 antagonism markedly reduces exosomal HMGB1 levels and improves survival (57). These findings suggest that a similar lactate–HMGB1 lactylation axis may participate in sustained inflammatory activation in DN.

In sepsis-associated AKI models, lactate induces HMGB1 lactylation in macrophages, promoting its extracellular release, which subsequently triggers neutrophil extracellular trap (NET) formation (58). Although direct evidence for this axis in DN-related neutrophils is lacking, similar lactate–macrophage–neutrophil crosstalk may contribute to inflammatory activation in DN. A high-lactate microenvironment can also suppress T-cell proliferation, reduce cytokine secretion, and increase the proportion of regulatory T cells. The immunosuppressive effect of lactate has been reported in multiple reviews of urological diseases (59). However, there is currently no direct evidence that links lactylation itself to T-cell regulation; thus, the immunosuppressive effect of lactate on T cells may not be attributed to lactylation-mediated epigenetic modification, but rather to the metabolic consequences induced by lactate accumulation.

### 4.5 Fibroblasts

Renal fibroblasts are the major effector cells mediating renal interstitial fibrosis (RIF) in DN. Their sustained activation and excessive extracellular matrix (ECM) deposition represent the key structural basis of DN progression. Recent findings suggest that the lactate–lactylation axis may serve as a previously underappreciated regulatory mechanism contributing to fibroblast activation and fibrotic remodeling.

Renal fibroblasts in chronic kidney injury models have been reported to display metabolic reprogramming; however, direct evidence for such alterations in fibroblasts from DN remains limited (23, 60). Rather than relying primarily on intrinsic PFKFB3-driven glycolytic flux, fibroblasts have been suggested to respond to lactate exported from neighboring tubular epithelial cells, which may enhance chromatin accessibility at profibrotic loci and promote transcriptional activation of ECM-related genes (23, 38), indicating a paracrine metabolic–epigenetic interaction between tubular cells and fibroblasts.

In addition to histones, research on non-histone lactylation remains limited. So far, no direct evidence has been provided to demonstrate specific non-histone lactylation targets in renal fibroblasts. However, findings from other systems suggest that lactylation may also modulate the stability and activity of transcriptional regulators such as HIF-1α and TFEB, or other metabolic enzymes through non-histone protein modification (61, 62). Similar non-histone lactylation events may plausibly occur within the high-lactate microenvironment of renal fibrosis, where they could influence fibroblast metabolism and transcriptional behavior.

### 4.6 Other renal cell types

Beyond the major cell types discussed above, several additional renal cells may also participate in lactylation-mediated injury, yet remain largely uncharacterized.

At present, there is no direct evidence demonstrating lactylation in glomerular mesangial cells in DN. Recent work in lupus nephritis has shown that excess lactate induces PBX1 lysine lactylation at K40, which drives mesangial cell proliferation, metabolic reprogramming, and extracellular matrix accumulation, thereby exacerbating glomerular injury (63). Because mesangial hyperproliferation and matrix expansion are shared pathological features of lupus nephritis and DN, these findings raise the possibility that a similar PBX1 lactylation–dependent program might also operate in diabetic kidneys; however, this currently remains a hypothesis and has not been experimentally verified in DN. This finding provides an important mechanistic clue suggesting that mesangial cells in DN may also be susceptible to lactate-driven epigenetic regulation, although direct confirmation is still lacking. Meanwhile, increasing attention is being paid to pericytes and other non-classical renal cell types. Current evidence suggests that metabolism of lactate in pericytes may influence capillary stability, peritubular perfusion, and interstitial remodeling; however, no studies have yet demonstrated histone or non-histone lactylation in these cells (38, 64).

In summary, studies on lactylation in various types of renal cells are still in their early stages. However, growing evidence suggests that lactylation may serve as a central mediator linking metabolic stress to epigenetic regulation within the multicellular pathological network of DN. To fully delineate cell type–specific lactylation sites, temporal dynamics, and microenvironmental coupling, future work will require integrated single-cell omics and spatial transcriptomics. Collectively, these insights support the concept that lactylation contributes to intercellular crosstalk and coordinates metabolic–epigenetic signaling across multiple renal cell populations.

## 5 Feedback amplification mechanisms of the vicious cycle

Building upon these findings, in DN, excessive lactate and its epigenetic consequences are not merely outcomes of metabolic reprogramming—they actively reinforce metabolic disturbances, forming a self-perpetuating metabolic–epigenetic vicious cycle. It should be emphasized that current evidence for a fully closed ‘lactate–lactylation–glycolysis’ loop in DN is still incomplete, and many mechanistic insights have been extrapolated from non-renal or non-diabetic contexts. Thus, these proposed amplification mechanisms require further validation in diabetic kidney tissues to clarify their relevance and specificity.

### 5.1 Positive feedback regulation of glycolytic genes by lactylation

Studies have shown that lactate, the end-product of glycolysis, can act as a metabolic signal and promote transcriptional activation of stress- and metabolism-related genes through histone lactylation. In pancreatic ductal adenocarcinoma cells, Li and colleagues found that H3K18 lactylation is enriched at the promoters of cell-cycle regulators such as TTK and BUB1B, thereby enhancing their transcription. The increased expression of TTK and BUB1B then feeds back to activate p300 and augment LDHA phosphorylation, raising lactate production and further increasing histone lactylation. This forms a self-reinforcing loop that links glycolytic flux, H3K18 lactylation, and promoter activation (65). A similar pattern was observed by Pan et al. in an Alzheimer’s disease model. They showed that H4K12 lactylation increases in microglia and accumulates at the promoters of key glycolytic enzymes such as PFKFB3 and ALDOA, thereby enhancing their transcription and amplifying glycolytic flux (66). These findings indicate that promoter-targeted lactylation may act as an epigenetic driver of metabolic activation across diverse pathological settings. Although such promoter-specific lactylation feedback loops have not yet been directly demonstrated in renal cells, both tubular epithelial cells and podocytes exhibit robust histone lactylation under diabetic or injury conditions, indicating that renal parenchymal cells possess the enzymatic machinery required for similar regulatory circuits. Thus, lactate-induced lactylation may amplify glycolytic gene expression and reinforce a metabolic feedback loop in DN, although this hypothesis requires kidney-specific experimental confirmation.

### 5.2 Dual mechanisms of lactylation-mediated stabilization of HIF-1α

HIF-1α is a central transcription factor controlling glycolysis and is normally degraded under normoxia through the prolyl hydroxylation–von Hippel–Lindau (VHL) ubiquitination pathway. Recent findings show that lactate accumulation can directly disrupt this degradation machinery, allowing HIF-1α to remain stable even in the absence of hypoxia. Li et al. demonstrated that HIF-1α undergoes lysine lactylation at specific residues (human K12 and mouse K644), which impairs its interaction with VHL, reduces K48-linked ubiquitination, and markedly enhances protein stability (61). This process establishes a “pseudo-hypoxic” metabolic state under normoxic conditions. Beyond stabilizing HIF-1α protein abundance, lactylation also augments its transcriptional potency. Lactylated HIF-1α exhibits enhanced recruitment to glycolytic gene promoters, leading to marked upregulation of downstream targets such as GLUT1, HK2, and LDHA (61). These findings reveal a dual amplification mechanism—stabilized HIF-1α abundance and enhanced transcriptional potency. Although primarily described in cancer, the persistent HIF-1α activation seen in DN suggests that a similar amplification may occur in renal tissues, though direct evidence remains lacking.

### 5.3 Inhibitory effects of lactylation on mitochondrial oxidative metabolism

Aside from its role in promoting glycolysis, lactylation has also been shown to directly impair mitochondrial oxidative metabolism; however, most supporting data originate from hypoxic or non-renal disease models, and its relevance to DN remains largely hypothetical.

Mao et al. reported that under hypoxic conditions, several mitochondrial proteins—including PDHA1 and CPT2—undergo lysine lactylation, which is accompanied by reduced OXPHOS efficiency and a metabolic shift toward glycolysis (67). Although the enzymatic consequences of individual lactylation sites were not tested, the global decline in mitochondrial respiration strongly suggests that mitochondrial protein lactylation exerts a suppressive effect on oxidative metabolism.

Lactylation is a reversible modification, and mitochondrial de-lactylation is mediated predominantly by SIRT3. Locatelli et al. showed that restoring SIRT3 activity reduces mitochondrial protein lactylation and enhances OXPHOS capacity, highlighting a direct link between lactylation status and mitochondrial energy homeostasis (68). These findings imply that dysregulated lactylation may contribute to mitochondrial vulnerability under diabetic conditions.

In the kidney, mitochondrial dysfunction has been closely associated with activation of the NLRP3 inflammasome in DN (69). Yu et al. further demonstrated that lactylation of citrate synthase (CS) can activate NLRP3 in another disease model (53), providing mechanistic insight into a possible “lactylation–metabolic stress–inflammation” pathway. Taken together, these findings suggest that lactylation-driven metabolic impairment may contribute to the amplification of inflammatory responses in DN. These findings suggest that lactylation-driven impairment of mitochondrial metabolism may not only compromise ATP production but also amplify inflammatory signaling, thereby exacerbating renal injury in DN.

### 5.4 Amplification effects of delactylase dysfunction

Lactylation is a reversible post-translational modification whose homeostasis is maintained by a dynamic balance between “writers” and “erasers.” Meanwhile, class I HDACs (HDAC1–3) and sirtuins—most notably SIRT3—have been reported to exhibit delactylase activity in foundational studies; however, renal and DN cell-type–specific validation remains limited (45, 46). In DN, chronic hyperglycemia and oxidative stress markedly reduce both the expression and enzymatic activity of these delactylases, creating a cellular environment that favors lactylation accumulation. As a mitochondrial deacetylase, SIRT3 is markedly downregulated in DN kidneys (68). Impaired SIRT3 function results in persistent lactylation of pivotal oxidative enzymes (PDHA1 and CPT2), thereby blocking pyruvate oxidation and fatty acid β-oxidation and disrupting mitochondrial ATP production (67). These effects are compounded by chronic hyperglycemia and inflammation, which further reduce intracellular NAD^+^ levels—the essential cofactor for SIRT1/3 activity, reinforcing delactylase dysfunction (64). Metabolomic and proteomic analyses have demonstrated widespread increases in lactylation sites in DN and db/db mouse kidneys, particularly within mitochondrial proteins (37, 64), indicating that delactylase inhibition is a major contributor to lactylation overload. Dysfunction of the delactylation system locks renal cells into a high-lactylation state, sustaining aberrant activation of pro-inflammatory and pro-fibrotic pathways even when upstream metabolic stimuli fluctuate (38, 49, 70). Pharmacological activation of SIRT3 or the supplementation of NAD^+^ precursors such as NMN can partially rescue delactylase function and alleviate mitochondrial injury. These findings suggest that reactivating delactylation represents a promising strategy to interrupt the metabolic–epigenetic vicious cycle in DN and to restore mitochondrial and transcriptional homeostasis (46, 68).

## 6 Translational prospects of lactylation in diagnosis and therapy

### 6.1 Detection techniques for lactate and lactylation

Strict distinction of lactate level and lactylation status is beneficial for understanding its role in DN. Traditional clinical lactate assays are rapid and accessible, but they provide only systemic measurements with limited cellular or spatial resolution. Thus, additional technologies are required to capture the cell type–specific and subcellular distribution of lactate and lactylation. Metabolomics enables quantitative profiling of lactate and its intermediates, offering insights into global metabolic alterations. More recently, single-cell and spatial metabolomics have emerged as powerful tools to map lactate distribution across nephron segments and renal cell populations (71). These approaches help bridge metabolic changes with spatial microenvironmental contexts in DN. In parallel, significant progress has been made in detecting lactylation itself. Antibodies specific for histone lactylation sites, such as H3K18la, have been developed and applied in immunohistochemistry (IHC) to visualize the spatial distribution of lactylation at the tissue level. In addition, ChIP-seq using lactylation-specific antibodies enables genome-wide profiling of lactylation-enriched promoters or regulatory elements (52), providing functional insights into lactylation-mediated transcriptional regulation. In summary, these technologies establish an integrated analytical framework that links metabolic alterations with epigenetic regulation. This framework lays the groundwork for developing lactylation-based diagnostic or prognostic biomarkers in DN, enabling more refined evaluation of renal metabolic–epigenetic states.

### 6.2 Correlation between lactylation levels and disease progression

Increasing clinical and experimental evidence support the notion that elevated lactate levels and heightened lactylation burden correlate with more severe DN phenotypes. However, a quantitative ‘critical’ lactate concentration or a validated lactylation-burden threshold beyond which renal injury becomes irreversible has not yet been defined in DN. Current clinical studies support an association (e.g., urinary lactate to creatinine ratio with eGFR decline) rather than a clear cutoff. In patients’ cohorts, greater systemic or tissue lactate burdens were accompanied by increased proteinuria, intensified interstitial fibrosis, and more rapid renal function deterioration (37, 38, 70). In non-diabetic kidney injury models, such as sepsis-associated AKI, elevated levels of histone lactylation marks (including H3K18la and H4K12la) have been reported to correlate with the severity of renal dysfunction (52). Whether comparable associations exist in human DN kidneys remains to be clarified, as systematic clinical studies are still lacking. In addition to histones, several non-histone lactylation events have also been implicated in DN pathogenesis. For instance, LARS1 is lactylated at lysine 970 in podocytes, and this lactylation impairs autophagy (50), while lysine lactylation of ACSF2 at K182 impairs mitochondrial metabolism and induces cell death in tubular epithelial cells (51). These novel lactylation events provide a molecular basis for the association between lactylation and phenotypic outcomes including podocyte loss, tubular atrophy, and interstitial fibrosis. Taken together, the existing evidence supports that lactylation may participate in DN pathogenesis as both a biomarker reflecting renal metabolic–epigenetic status and an active driver promoting DN progression.

### 6.3 Therapeutic strategies targeting lactylation

As lactylation is a reversible post-translational modification, therapeutic interventions in DN may follow two principal strategies: approaches that reshape upstream metabolism and thereby influence lactylation secondarily, and approaches that aim to modulate lactylation more directly. Both approaches aim to disrupt the metabolic–epigenetic feedback loop that sustains renal injury.

On the one hand, several interventions already used clinically—or widely discussed in DN—may indirectly affect metabolic reprogramming and the glycolysis–lactate axis. Experimental studies have shown that inhibition of key glycolytic enzymes such as LDHA or PFKFB3 lowers lactate levels and, in turn, reverses proteinuria and renal fibrosis in mouse models (24, 72). These findings highlight the therapeutic potential of targeting upstream metabolic flux. Furthermore, SGLT2 inhibitors, by lowering glucose delivery to proximal tubular cells and suppressing glycolytic flux, may theoretically decrease lactate availability and thus influence downstream lactylation dynamics; however, direct evidence in DN models is still lacking. Metformin, through modulation of cellular energy sensing (e.g., AMPK-related pathways) and mitochondrial metabolism (73), may also indirectly reshape the glycolysis–lactate axis; however, whether such upstream metabolic effects translate into measurable changes in renal lactylation in DN remains to be determined.

On the other hand, a more direct strategy is to intervene in the lactylation machinery itself. For example, pharmacological inhibition of p300/CBP has been reported to reduce histone lactylation and repress transcription of pathogenic genes in relevant experimental settings (7, 74). In contrast, activation of delactylases like SIRT1 or SIRT3, or the supplementation of the NAD^+^ precursor NMN, may enhance delactylation and, in turn, may help restore the metabolic–epigenetic balance, enhance mitochondrial function, and protect the kidney (45, 46).

However, feasibility and safety considerations are central, given lactate’s essential physiological roles and the broad substrates of many candidate epigenetic enzymes. A key translational challenge is to selectively dampen pathological lactylation in the diseased kidney without disrupting systemic metabolic homeostasis or causing non-specific transcriptional effects. Despite these promising findings, several challenges remain. Cell type–specific delivery will be essential to avoid off-target effects when modulating metabolic or epigenetic pathways. Additionally, *in vivo* tools capable of precisely modulating and monitoring lactylation dynamics are still under development. These limitations highlight the need for refined technologies to support the clinical translation of lactylation-targeted therapies.

### 6.4 Translational opportunities

Building upon these mechanistic advances, the emerging concept of lactylation as a crosstalk between metabolism, epigenetics, and organ pathology offers several promising translational opportunities. From a clinical perspective, lactylation-based biomarkers may enable earlier diagnosis and improved risk stratification in DN. For instance, patients with an increased lactate-to-pyruvate ratio or elevated H3K18la levels could represent a high-risk subgroup prone to rapid disease progression. Meanwhile, therapeutic modulation of both metabolic fluxes—such as glycolysis and mitochondrial pathways—and epigenetic regulators including p300 and SIRT proteins may help disrupt the self-perpetuating “lactate–lactylation–glycolysis” loop that drives renal injury.

In parallel, the rapid progress of spatial metabolomics and epigenetic imaging technologies has made it possible to map lactylation patterns at the cell type–specific level within the kidney, providing a foundation for precision metabolic and epigenetic therapy. Nevertheless, several challenges must be addressed before these translational opportunities can be realized. These include the standardization of quantitative lactylation assays, the identification of safe and selective pharmacological modulators, and the development of tools for dynamic *in vivo* lactylation imaging. Looking ahead, integrating metabolic interventions (e.g., SGLT2 inhibitors or glycolysis blockers) with epigenetic therapies (such as p300 inhibitors and SIRT activators) may usher in a new “metabolic–epigenetic” era in the treatment of DN.

From a translational perspective, lactate- and lactylation-related readouts may have clinical value for risk stratification and on-treatment pathway monitoring, but their utility will depend on kidney specificity and assay standardization. Systemic lactate is accessible yet non-specific; therefore, combining lactate with renal-contextual metrics (e.g., urinary lactate normalized to creatinine, lactate/pyruvate as a redox proxy) and, when kidney tissue is available, assessing histone lactylation markers (e.g., H3K18la or H4K12la) may help reflect intrarenal metabolic–epigenetic activity, although current evidence in DN remains limited and part of the support comes from kidney injury models (37, 52). Mechanistically, this framework also supports a realistic therapeutic logic: early-stage interventions that reduce proximal tubular glucose load and glycolytic pressure (e.g., SGLT2 inhibition or upstream metabolic modulation) may limit lactate availability, whereas later-stage disease dominated by hypoxia, inflammation, and fibrosis may require combining metabolic modulation with downstream epigenetic rebalancing (e.g., targeting lactylation-related enzymes or enhancing delactylation capacity), ideally in a kidney- or cell-informed manner to minimize systemic metabolic disruption.

## 7 Limitations and future directions

The identification of the “lactate–lactylation axis” offers a new paradigm for understanding the relationship between metabolism and epigenetics in DN, but its clinical application remains in infancy. Most of the current evidence comes from cell and animal studies, whereas longitudinal studies using human samples are few and far between. Future work should move beyond correlations to establish mechanistic causality between defined lactylation events and DN progression, particularly to determine whether lactylation is an early pathogenic trigger or, more plausibly based on current evidence, a downstream amplifier that stabilizes metabolic and inflammatory injury programs. Notably, several mechanistic components of this model originate from non-diabetic or non-renal disease systems. In addition, clinical trials that specifically target lactylation-related pathways in DN are currently lacking, which limits evidence-based assessment of feasibility, safety, and efficacy in patients.

The technical challenges of developing sensitive, reproducible, and clinically applicable assays for lactylation detection remain a bottleneck to translating lactylation biomarkers from bench to bedside. Safe and controllable methods to modulate lactylation in patients also remain unavailable. Accordingly, some therapeutic proposals discussed in this review should be regarded as hypothesis-generating until they are validated by DN-focused *in vivo* studies and, ultimately, clinical investigation. Although controlled modulation of lactylation has been demonstrated *in vitro*, the use of advanced single-cell and spatial omics technologies to understand cell-type-specific patterns of lactylation is an emerging reality. Owing to recent advances in small-molecule drug development targeting epigenetic enzymes, it is becoming increasingly feasible to discern the dynamic and functional characteristics of lactylation across different cell populations and disease stages.

A major challenge is how to translate the findings from these mechanistic studies into clinically actionable therapies. This will require integrated, cross-disciplinary collaboration among metabolism, epigenetics, and nephrology experts in the context of systems biology and precision medicine. Overcoming these challenges will be the determining factor in whether the lactylation field can successfully bridge the gap from molecular mechanisms to clinical application and thereby open new therapeutic avenues for DN.

## 8 Conclusion

Metabolic reprogramming and lactylation are key molecular mechanisms in DN. The progression of DN is closely related to the metabolic transformation of renal cells, especially the shift from mitochondrial oxidative phosphorylation to enhanced glycolysis. This metabolic alteration leads to the accumulation of lactate and affects the transcription programs through lysine lactylation, providing a mechanistic bridge between glycolytic flux and chromatin-associated regulation. Importantly, lactylation should be viewed as a promising yet still emerging epigenetic mechanism in DN, with the potential to reshape how DN progression is conceptualized as an integrated metabolic–epigenetic process.

These findings provide a new molecular mechanism framework for DN, revealing the interaction between metabolism and epigenetics. In terms of therapeutic prospects, strategies that modulate upstream metabolic stress and downstream lactylation-related signaling may offer new directions for early intervention and risk stratification in DN. However, many proposed targets remain supported primarily by experimental models, and direct validation in human DN and clinically relevant settings is still limited. Future research should therefore prioritize DN-focused causal evidence linking defined lactylation events to pathogenic transcriptional programs, standardized and quantitative detection approaches suitable for translational use, and kidney-/cell-informed intervention strategies that balance efficacy with specificity and safety. Only with rigorous experimental validation and careful translational development can lactylation-guided biomarkers and therapies move toward clinical application in DN.
